# Testing semantic compositionality in baboons (*Papio papio*) through relearning and generalization

**DOI:** 10.1371/journal.pone.0334726

**Published:** 2025-11-05

**Authors:** Anne Reboul, Nicolas Claidière, Isabelle Dautriche, Joël Fagot

**Affiliations:** 1 Centre de Recherche en Psychologie et Neurosciences, CNRS UMR, Aix-Marseille Université, Marseille, France; 2 Station de Primatologie-Celphedia, CNRS UAR, Rousset, France; Tshwane University of Technology, SOUTH AFRICA

## Abstract

This study investigates whether baboons are capable of semantic compositionality, specifically, whether they can apply compositional rules to new situations (generalization). In language, semantic compositionality is linked to productivity, the generalization of a rule to new combinations. Across four experiments, baboons were trained to match visual stimuli based on either shape or color depending on symbolic cues. Experiments 1–3 tested generalization under different task complexities but consistently failed to show evidence that baboons understood or applied the matching rules beyond memorized combinations. Only in Experiment 4, which used a relearning paradigm rather than generalization, did baboons show improved performance when the rule remained consistent across phases. Four hypotheses were explored to explain the lack of generalization: an iconicity-novelty bias, the possibility that compositionality is present, but that training was not sufficient for generalization, rote memorization of cue-sample pairs, and a difference between implicit and explicit learning. The findings do not allow us to discriminate between these hypotheses.

## Introduction

A central feature of human language is semantic compositionality. In semantic compositionality, the meaning of a sequence of words is dependent on the meaning of its constituents. Additionally, the meaning of the whole is determined by semantic rules. Semantic rules link the structure of the sequence and the meaning of its constituents to the meaning of the whole sequence. The structures concerned can vary from simple juxtaposition (e.g., “brown cow” that refers to anything that is both a cow and brown) to more complex constructions (e.g., “not brown cow” that refers to anything that is a cow and not brown, or to anything that is neither a cow nor brown). This leads to a distinction between trivial semantic compositionality, where two information are combined, but may come from different sources and are not structurally organized, and non-trivial semantic compositionality, where two information come from the same source and are structured in a single sequence. One hallmark of semantic compositionality in human language is productivity. Once the rules for a given pattern of semantic compositionality are acquired, it is possible to produce an unlimited number of sequences following the same pattern (for instance, once the rule for interpreting “brown cow” are understood, it is possible to produce any number of similar constructions, e.g., “red car”, “blue shirt”, “tall woman”, “beloved child”, “even number”, and so on). In other words, while there could be compositionality without productivity, productivity is a strong indicator of compositionality.

While field studies (studying nonhuman animals in the wild) have noted some sequential signals incorporating two calls or more in animal communication systems [1–10], there is no evidence of productivity in these studies. This might be because of the limited size of the set of signals used in animal communication systems. Or it might be due to failure to generalize. Additionally, there is no convincing evidence either that these are instances of semantic compositionality rather than idiomatic meaning in nonhuman primates (for a debate, see [1,11–13]). Through a series of experiments, Suzuki et al. [6–8] provided evidence for semantic compositionality in Japanese tits. Schlenker and colleagues [13] reviewed these studies and found the results to be compelling. Thus, there is limited evidence for non-trivial semantic compositionality in animal communication systems from field studies, but none so far in nonhuman primates. And there is no evidence of productivity in cases where arguably there is semantic compositionality.

Experimental studies, investigating the ability for semantic compositionality in nonhuman primates have not fared much better [14–16]. There is one exception, however. In 2022, Dautriche and colleagues [17] used negation as an example of semantic compositionality and were able to show that baboons can master a form of negation by choosing the complement target when a specific index is added to the cue. A limit of this study, however, is that the test was based on relearning rather than on generalization, and, arguably, generalization would be necessary for productivity. One may think that relearning goes with generalization, as, if there is generalization, relearning would be strongly facilitated, but this point remains to be demonstrated. Nevertheless, to our present knowledge, semantic compositionality seems difficult for nonhuman primates, suggesting that semantic compositionality in humans depends on language or is at least strongly facilitated by language. However, this might be a result of the difficulty of devising the right experimental paradigm to test nonhuman animals on semantic compositionality.

In the present study, we chose to test baboons’ capacity to match a visual sample to a visual target with either a similar color or a similar shape, based on two different cues (one for color, one for shape). During testing, baboons were presented with the same cues (indicating that they should match either on color or shape), but with items with new colors and new shapes that they hadn’t seen before. They had to match them successfully to the target. Generalization was therefore tested with this design. The goal was to ascertain whether an ability for this type of semantic compositionality could be evidenced experimentally in nonhuman primates. Semantic compositionality is linked in humans to the ability to generalize cues to new stimuli. Therefore, positive results would show that one condition for productivity, generalization, is available in nonhuman primates.

To cut a long story short, Experiment 1 did not yield the results we hoped for, and we did three follow-up experiments to try and understand why the baboons failed to generalize in this first experiment. Here is a synthetic table of the sequence of experiments (a procedural table for all four experiments can be found in Supporting information, S1 Table):

## Experiment 1

### Materials and methods

#### Participants and living conditions.

The study was conducted on 23 Guinea baboons (*Papio papio*) from the CNRS Primate Centre in Rousset-sur-Arc (France), mean age at the onset of the study 13.25 years, min = 4.9, max = 24; 7 males). The baboons belonged to two social groups, one comprising 6 males and 12 females, and another with 1 male and 4 females. The larger group was housed in group in a 700m^2^ outdoor enclosure connected to two 24m^2^ trailers equipped with the test systems (see below) and an indoor enclosure. The smaller group was housed in a 24m^2^ outdoor enclosure connected to a 18m^2^ trailer containing additional test systems and an indoor enclosure. Outdoor enclosures feature various enrichments, such as climbing structures or stones that the baboons can manipulate. The indoor enclosure was equipped with platforms used at night. Baboons were neither water nor food deprived. They received their daily food ration at 4 pm (fruits, vegetables, and monkey chows), and water was provided ad libitum within each enclosure. All baboons were familiar with the matching-to-sample (MTS) procedure, due to previous testing (e.g., [18]), but the test was only proposed to the subset of subjects who successfully passed the learning criterion (see below).

#### Animal welfare.

Our research used Automated Learning Devices for Monkeys (ALDM) which have been described in detail in [19–20]. As described below, these test systems make it possible to test the individuals without separating them from their social group. Earlier studies from our research group have shown that this procedure reduces the stress level during the experiment, as demonstrated by reduced cortisol levels, as well as the frequency of stereotypies [21]. Daily observations of the baboons by the animal care staff of the CNRS primatology station and a dedicated ethologist guaranteed animal welfare during the experiment. Any sign of discomfort, injury or sickness during the experiment triggered a health check by the animal care staff. This study involved no anesthesia, analgesia, or euthanasia. The subjects stayed in their home social groups during the research and remained in their social group after completion of the experiment.

#### Apparatus and general test procedure.

This research used a total of 14 ALDM test systems (n = 10 for the large group, n = 4 for the smaller group) which were installed within the trailers along the enclosures and were accessible through holes made in the wire mesh. In short, each ALDM comprised a RFID (Radio Frequency Identification Device) reader for the identification of the baboons, a 19-inch touch screen for stimulus presentation and the recording of behavioral responses, and a food dispenser delivering a food reward (a drop of dry wheat) within the ALDM cubicle after each correct response to the task. The baboons had a biocompatible 1.2 by 0.2 cm RFID microchips implanted in each forearm and were automatically identified by the RFID readers whenever they entered an ALDM test chamber. The automatic identification served to resume the trial list at the place at which the subject left it at its previous visit in the same or a different ALDM. This procedure enabled the baboons to engage with the test design in a context where they could choose to participate or quit testing at will, while remaining in their social group, without the need to capture them.

The experiments presented in this paper all used a zero-delay MTS procedure. In practice, identification of the subject in the test system triggered the appearance of a fixation point at the bottom center of the screen, which the baboons had to touch. This action displayed the sample stimulus in the upper center of the screen. Touching the sample, in turn, displayed two choice stimuli, one on the left and one on the right bottom part of the screen. No delay was introduced between the sample and comparison stimuli. The baboons had to touch the target stimulus defined by the matching rule (which differed from test conditions, see below). Correct responses were followed by the delivery of a drop of wheat serving as reward. Incorrect responses were followed by a 3 sec time-out with a green screen, indicating that an error has been made. All stimuli were 180X180 pixels and were displayed on a black background. The experiments were controlled by a software testing program written by JF using E-prime (Version 2.0 professional, Psychology Software Tools, Pittsburgh, PA, USA).

#### Stimuli and test procedure.

**Fig 1:**
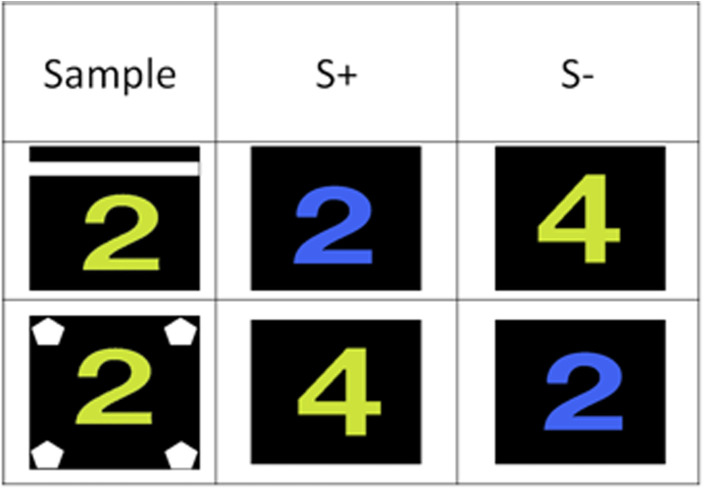
Experimental design of Experiment 1. This figure shows the prototypical design of the matching trials. The baboons had to match on shape when a horizontal bar was added to the sample stimulus, and on color when small pentagons were added to the sample.

This experiment used a total of 100 different stimuli made from the combination of 10 colors and 10 (letter or number) shapes. Fig 1 illustrates the matching rule to be learned in that experiment: in each trial, the target stimulus shared a single dimension with the sample, for instance its color, while the foil stimulus shared the alternative dimension, thus its shape in that example. Form trials were indicated by the display of a horizontal bar above the sample. Color trials were indicated by four small pentagons surrounding the sample. To be rewarded, the baboons had to select the comparison stimulus matching the sample regarding the cued stimulus dimension, either the form or the color.

Testing in Experiment 1 was organized in 9 phases. Phase 1 served to teach the matching rule to the participants. It only used four stimuli made from two colors and two shapes. Phase 1 was organized in 120 trial blocks comprising 60 form trials randomly intermixed with 60 color trials. This criterion corresponds to a performance beyond chance level (one-tailed binomial test, p < .05) for each category.Once this criterion was reached, the baboons automatically moved to the next phase.

Phases 2–9 followed the same logic as Phase 1, except that we added one novel color and one novel shape to the list of colors and shapes used in the previous phase. We therefore combined 3 shapes and 3 colors to create 9 different stimuli in phase 2, 4 shapes and 4 colors to create 12 stimuli in phase 3, and this procedure was followed until phase 9 (10 colors and 10 shapes to create 100 different stimuli). This procedure allowed distinguishing four types of trials. They were the “old color” and “old shape” trials in which the color and shape of the sample was selected from those used in the previous phase, and the “new color” and “new shape” trials, where the sample used the novel color and/or the novel shape introduced in the phase. In all trials, the target stimulus shared its cued dimension with the sample, for instance its form in form trials. The foil shared the non-cued dimension with the sample, i.e., its color in this example. The alternative dimension of the target and foil stimuli was selected from the full set of colors and shapes used in the phase (see Fig 1). The trial blocks of phases 2–9 combined 30 trials of each trial type (120 trials altogether). The criterion was set at 66% correct, corresponding to above-chance performance according to a one-tailed binomial test (p < .05). This threshold was deemed optimal for training, as participants had to reach 66% in all four training conditions to be considered trained in the condition, and progress to the next one.

#### Statistical analyses.

The goal of statistical analyses was to determine whether the baboons understood the matching rule: match on color in the presence of a line cue and on shape in the presence of pentagon cues. The alternative strategy was their reliance on rote memory (memorizing the response for each stimulus/cue combination), or other unforeseen strategies. We predicted that if they learned the matching rule then (1) the number of blocks they needed to reach criterion would decrease from phase to phase (it would not if they rely on rote memory), (2) their average success rate in the first block of a new phase would be higher than chance for the newly introduced stimuli (i.e., they would generalize; alternatively it should be 50% if they rely on memory) and (3) their average success rate on previously learned stimuli should not decrease (whereas it should become harder and harder to remember all the stimuli by rote).

To analyze performance, we used lme4 package in R to perform binomial generalized linear mixed models (GLMM) with a logit link function. The identity of the individual was used as random factor.

#### Ethical statements.

This research adhered to French and E.U regulation for the ethical treatment of research animals. It received ethical approval from the National French ethics committee « Comité d’Ethique CE-14 » for experimental animal research, as well as the French Ministry of Education (approval APAFIS#2717−2015111708173794 10 v3).

### Results

Of the 23 monkeys that participated in the experiment, 15 passed the criterion in phase 1, ten passed it in phase 2, six passed phase 4, and only one (Feya) passed the criterion in phase 8 (Table 1). Looking at the 10 monkeys that passed the criterion at least twice, we found evidence of a decrease in the number of blocks to reach criterion (Spearman correlation test, N = 10, S = 14563, p-value = 0.02, ρ = −0.37), but this was largely linked to a decrease in the number of blocks between the first and the second phase, which could be explained by monkeys simply learning to do the task. There was no significant evidence of a similar trend for the six monkeys that passed the first four phases (N = 6, S = 5179, p-value = 0.15, ρ = −0.28), nor for Feya alone that passed eight phases (N = 1, S = 46, p-value = 0.27, ρ = 0.45) Table 2.

**Table 1:** Synthetic presentation of the four experiments, their rationale and results.

	Goal	General principle	Predictions	Results	Interpretation
**Experiment 1**	Extend Dautriche et al’s [17] results, by testing generalization with a design using two cues, one for color and one for shape.	Progressive increment of the number of colors and forms. Generalization is measured by baboon’s response to novel stimuli.	Baboons will progressively learn to generalize the matching rule to novel forms and novel colors	Baboons failed to generalize the matching rule, or even relearn the response rule faster, even after nine training phases.	Use of two cues might be too demanding for baboons
**Experiment 2**	Test generalization in a matching task involving a single cue: the cue requires the choice for the stimulus that differs in color from the sample	Progressive increment of the number of colors and forms. Generalization is measured by baboon’s response to novel stimuli.	Reduction of the number of cues to one should promote generalization	No evidence for generalization	Use of two stimulus dimensions (color and shape) might be too demanding for baboons
**Experiment 3**	Test generalization in a matching task involving one single cue, as in Exp 2. The comparison stimuli only differ in color (test1), or in both color and shape (test 2).	After an initial training to criterion with a first stimulus set only differing in color, generalization is assessed in test 1 with novel colors, and in test 2 with novel colors and novel shapes	The simplification of the task should promote generalization	No evidence for generalization in neither test 1 nor test 2	We are unable to replicate Dautriche et al’s [17]. findings in Exp. 1–3. Relearning must therefore be tested to confirm their results.
**Experiment 4**	Test relearning instead of generalization, as in Dautriche et al. [17]	Baboons are exposed to two test phases involving novel stimuli, but for which the rule can either be identical or opposite to training. The dependent variable is leaning speed	Relearning should be facilitated when the rule is the same as in training.	Relearning is significantly faster when the rule is consistent to training	Exp. 4 confirms Dautriche et al. [17] conclusions of compositionality, but this ability is only shown through relearning.

**Table 2:** Number of blocks realised by each subject per phase of Experiment 1 (60 trials for phase 1, 120 otherwise). For instance, ARIELLE realised 28 blocks of 60 trials before reaching criteria and moving to phase 2 of the experiment. Note that the number of times the criterion was reached by a subject is the last experimental phase minus one (e.g., eight for FEYA). The means refer to the grand mean for the subjects that passed the criterion at least once (N = 15) or four times (N = 10).

	Experimental phase									
	1	2	3	4	5	6	7	8	9	Total
ANGELE	99									99
ARIELLE	28	49	10	6	88					181
ARTICHO	14	25								39
ATMOSPHERE	100									100
BOBO	29									29
DORA	68	35								103
DREAM	26	18	25	6	59					134
EWINE	77	25	124	109						335
FANA	61	34								95
FELIPE	14									14
FEYA	22	14	7	5	19	29	16	63	48	223
FLUTE	60									60
HARLEM	18									18
HERMINE	38									38
LIPS	71	11	26	87						195
LOME	33	36	7	73						149
MAKO	45	21	44	25	3	59				197
MALI	48	35	167							250
MUSE	38	37	38	66	16					195
NEKKE	162	11								173
PETOULETTE	99	27								126
PIPO	20									20
VIOLETTE	46	14	244	8	91					403
Mean criterion at least once (n = 15)	55,9	26,1	46,1	25,7	18,4	5,9	1,1	4,2	3,2	187
Mean criterion at least four times (n = 6)	34,2	25,5	61,3	19,3	46	14,7	2,7	10,5	8	222

Regarding the average success rate in the first block of a new phase for individuals that passed at least two training stages (N = 10), we found a small increase in average success with the number of phases for new colors (GLMM: β = 0.13, SE = .04, z = 2.95, p = 0.003), but none for new forms (β = 0.03, SE = .04, z = 0.63, p = 0.53). Surprisingly, on the first block of phase 2, the average score was already above chance for new colors and new shapes (colors: β = 0.51, SE = .14, z = 3.61, p < 0.001; shapes: β = 0.89, SE = .14, z = 6.21, p < 0.001). Success rate was estimated to be 62% (95% CI = [56%; 69%] on average for colors and 71% [65%;76%] on average for shapes. This, however, was linked to low scores on previously learned colors and shapes in the same first blocks (estimate for block two were 52% [47%, 57%] for colors and 49% [44%, 54%] for shapes), despite monkeys reaching an average 66% correct on the same stimuli in the block just preceding.

**Fig 2:**
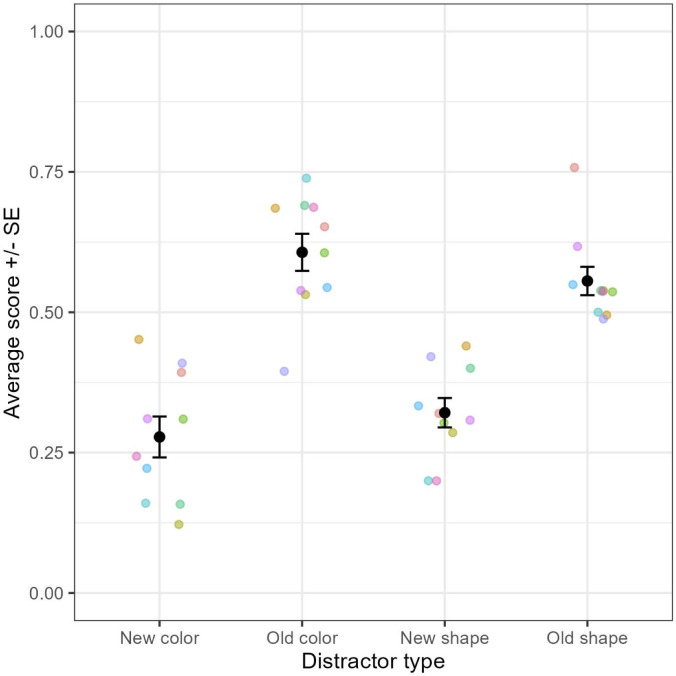
Average success rate in Experiment 1, all phases combined, when the target is known, and the distractor contains new vs. old dimensions. Each colored dot represents the average score for one subject. Black dots and error bars represent the grand average + /- standard error.

At first puzzling, this situation became clearer when we realized that when presented with a previously learned stimuli, baboons had very low scores when the distractor contained a newly introduced color or form (Fig 2). All phases included, when matching on color with a previously learned sample and distractor, the average score was 61% [54%, 67%] but fell to 28% [21%; 35%] when a distractor contained a new dimension (form or color or both). Similarly, when matching on shape with a previously learned sample and distractor, the average score was 56% [51%, 61%] but dropped to 32% [27%; 37%] when a distractor contained a new dimension (form or color).

Taken together, our results are compatible with a memory-based strategy consisting of memorizing the correct response for each combination of stimulus and cue, independently, combined with a bias for selecting the new shape or new color when it appears. Given that half of the trials consisted in the new shape or color and the remaining half of all the old shapes and colors, the strategy consisting of systematically selecting the new dimensions produced a few mistakes when they were used as distractors, and an above average success when they were used as sample (on average the ten monkeys that passed two phases maintained a score of 68%). In any case, there was no sign of understanding of the meaning of the symbolic cues in this experiment.

### Interim discussion

In Experiment 1, we investigated the capacity of baboons to use cues to match either on the form or on the color of the sample. The test was generalization. The results were disappointing as baboons failed to generalize. While the experiment did not test relearning, it is interesting to note that the number of blocks necessary to pass from one phase to the next did not decrease in the monkeys that completed 4 phases. Nor did it in the only monkey (Feya) that passed 8 phases. Thus, there is no indication of relearning either, in contrast to Dautriche and colleagues [17].

One possible explanation for the failure of the monkeys to understand the rule is the number of stimulus dimensions involved. In this experiment, they had to pay attention to the color and to the form of the sample, as well as to two different cues. This task is therefore more demanding than [17], which used only one cue and one stimulus dimension in their test phase. Experiment 2 investigated whether the baboons would perform better if they only had to process one single cue, instead of two.

## Experiment 2

### Materials and methods

#### Participants, apparatus.

Experiment two used the same apparatus as above, and the same group of baboons. However, one of the previous baboons was not available for testing due to sickness. Data were therefore collected on 22 baboons in this second experiment. Twelve months elapsed between the end of Experiment 1 and the onset of Experiment 2, increasing the baboon’s age by one year in this second experiment.

#### Preliminary testing to determine initial dimensional (color or shape) biases.

This preliminary study examined if the baboons had a preference to match on color or shape, when they had the choice between the two options. To do so, the baboons were firstly MTS trained using four colored shapes as stimuli, which were built from two possible colors (yellow or orange) and two shapes (α or β). During training, the target stimulus was identical in color and shape to the sample, and the foil stimulus neither shared its shape nor its color with the sample. Training blocks comprised 64 trials fully counterbalanced regarding the identity of the sample and the position (left or right) of the target stimulus on the screen. These blocks were repeated until the baboons reached 80% correct. Reaching the learning criterion required 2.39 blocks on average (range 2–4).

Once learning criterion was reached, the baboons were exposed to 72 trial-blocks, comprising 64 trials identical to training and 8 test trials where one comparison stimulus had the same color as the sample but a different form, and the other one had the same shape but a different color. These test blocks used the same stimulus set as for training. Test trials within each block were randomly reinforced on a 80% basis, irrespective of the baboons’ response. This procedure allowed us to keep a rate of reinforcement identical to training, while not biasing baboons responses during test trials.

Because the number of test trials within a block (n = 8) remained minimal for statistical analyses, the test blocks were repeated five times to obtain a total of 40 test trials per baboon, and thus enhance statistical power. Results of this preliminary testing are given in the supporting information S2 Table. The results indicate a reliable bias to choose the color dimension. This bias was present in all but two subjects (i.e., Dream and Atmosphere who expressed no significant preference bias).

#### Training procedure.

**Fig 3:**
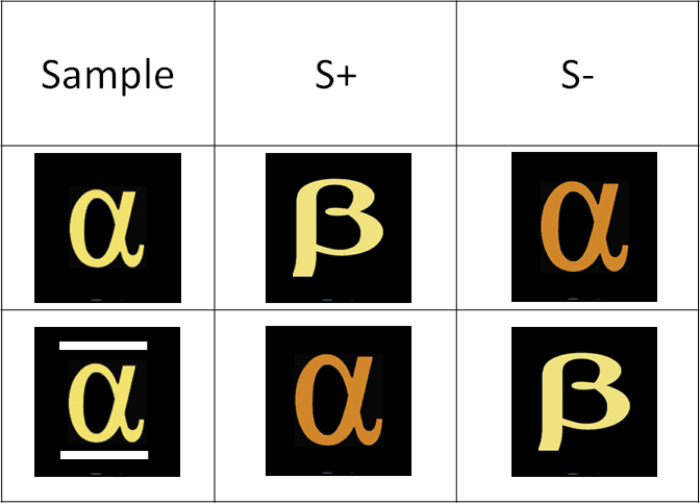
Task and experimental design (Experiment 2). This figure shows a prototypical stimulus arrangement during training. The baboons had to match on color in absence of cue (middle line), but match on the alternative stimulus when the negation cue is shown (bottom line).

Having determined the initial dimensional biases of the baboons, we next trained them to conditionally match the stimuli on color or shape, depending on the absence (match on colors) or presence of a symbolic cue (match on shape). This training used the same four stimuli as above arranged into MTS trials. In each trial, one response stimulus matched the sample only in color, while the other one only matched it in shape. Two kinds of training trials were proposed (Fig 3). In the no-cue training trials, the sample was displayed alone, without any symbolic cue. In that case, the baboons were required to choose the target stimulus matching the sample on a color basis. These trials were designed to align with the baboons’ initial dimensional bias for matching on color. A symbolic “negation” cue was added to the sample in symbolic no trials. That cue corresponded to the addition of two-line segments (one above and one below) to the sample, signaling to the baboons that they now had to inhibit their initial matching bias to select the alternative comparison. Said differently, the symbolic cue acted as a negation cue in the sense of [17]. The training blocks contained 64 trials, all food reinforced when the response is correct, in which the identity of the sample, left/right location of the target, and the cueing condition (color vs symbolic “no”) were counterbalanced. Training blocks were repeated until the baboons reached 80% correct in the two cueing conditions.

#### Test procedure.

This testing assessed if baboons could generalize the rule learned during training, when they are exposed to novel stimuli. Baboons were exposed to a single test block of 768 trials, comprising 640 baseline trials identical to training (10 repetitions of the earlier training blocks) intermixed with 128 test trials. The test trials employed 64 novel stimuli made from the combination of 8 novel colors and 8 novel shapes. Each novel test stimulus was repeated twice as sample within the test block to counterbalance the location of comparison stimuli on the left or right hemi-screen. The test trials were reinforced on a 100% basis to avoid biasing the baboon’s response in these trials. Baseline trials were food reinforced in case of a correct response.

#### Statistical analyses.

To analyze the results, we used lme4 package in R to perform binomial generalized linear mixed models (GLMM) with a logit link function. The identity of the individual was used as random factor, and the cue as a predictor variable. We analyzed separately the results for the baseline and test conditions to evaluate the performance of individuals depending on the presence or absence of the cue.

### Results

Twenty-two baboons were exposed to the training, but only 11 (2 males, mean age 11.9 years, range 2.2–17.8 years) successfully reached the training criterion. Learning the task required 74.9 training blocks on average (SD = 27.6, range 26–118).

**Fig 4:**
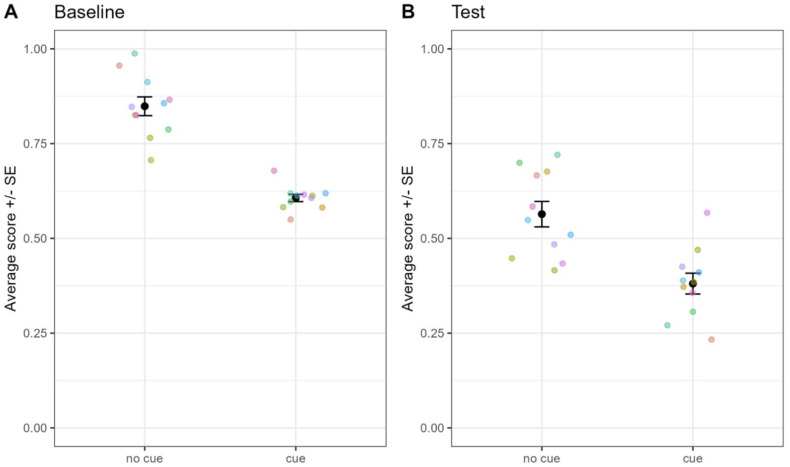
Performance depending on the absence (no cue: match on color) or presence of cue (match on shape) for the baseline (A) and test trials (B). Each colored dot represents the average score for one individual. Black dots represent grand average and error bars the standard error.

We found that during baseline trials (Fig 4), performance in the absence of cue (match on color) was significantly higher than with cue (match on shape; GLMM: β = −1.30, SE = .06, z = −22.2, p < .001), and both were above chance levels (no cue: β = 1.74, SE = .08, z = 22.0, p < .001; cue: β = 0.43, SE = .07, z = 6.06, p < .001). During test trials however, performance in the absence of cue was still significantly higher than with cue (β = −0.74,SE = .11,z = −6.85,p < .001), but the performance without cue was above chance (β = 0.25,SE = .08,z = 3.38,p < .001) and with cue below chance (β = −0.49,SE = .08,z = −6.27,p < .001). This suggests that individuals matched on color both with and without cues.

### Interim discussion

In Experiment 2, once a preference was established, the monkeys had to learn a rule which directed them, in the presence of a cue, to match the sample on the non-preferred dimension. Again, the baboons showed no sign of generalization, suggesting that they failed to understand the rule. It can therefore be concluded that reducing the number of cues to one did not help in this experiment.

## Experiment 3

Experiment 3 investigated whether baboons would do better if presented with stimuli that differed only in one dimension (color) rather than in two dimensions (form and color).

### Materials and methods

#### Participants, apparatus and stimuli.

Experiment 3 was proposed to the same 22 baboons which were tested with the same apparatus as in Experiment 1.

#### Training and test procedures.

**Fig 5:**
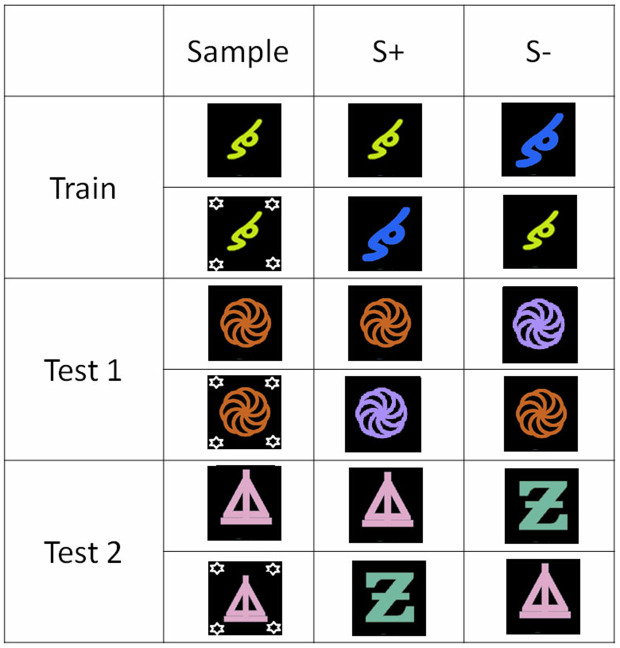
Task and experimental design used in Experiment 3.

Experiment 3 consisted of one training phase followed by two testing phases (test phases 1 and 2) presented in succession. Fig 5 illustrates the trial design of each phase.

The training phase followed the same general procedure as the training in Experiment 2, but it differed from it in two respects. First, the target and foil stimuli used within a trial only differed in color, and no more in both shape and color (Fig 5). Second, the negation cue was now composed of little stars surrounding the sample. Training blocks consisted of 48 cued and 48 non-cued randomly ordered trials (96 trials per block) involving a counterbalanced use of four stimuli made from two shapes and two colors. The training blocks were repeated until the baboons reached 80% in both conditions of cueing.

Test 1 only consisted of one block of 1104 trials, including 960 baseline trials randomly intermixed with 72 test trials. Baseline trials were identical to training and thus used the same four stimuli and same stimulus arrangement as in training. The test trials also used the same stimulus arrangement, but a novel set of 36 stimuli made from 6 novel colors and 6 novel shapes (Fig 5). Each novel stimulus was used twice as sample during Test 1 for counterbalancing the location of the target on the screen.

Test 2 primarily differed from Test 1 in terms of stimulus arrangement. In Test 2, the sample was identical to the target stimulus on both dimensions (shape and color), and the foil varied in both shape and color (Fig 5) from the sample. Further, Test 2 used a novel set of 36 stimuli made from 6 novel colors and 6 novel shapes different from those of the training and Test 1 stimuli.

Baboons were exposed to a matching-to-sample task, where they had to select a target stimulus whose identity depended on the presence (or absence) of a cue (four little stars) surrounding the sample. In the training phase, all the stimuli used within a trial had the same shape, and the identity of the target stimulus was only determined by its color (identical or different from the sample, depending on the cue). Test 1 used the same stimulus arrangement as in training, but a different set of stimuli made from new shapes and new colors. Test 2 also used a new set of stimuli made from other new shapes and new colors. However, in test 2, the two choice stimuli differed on both dimensions (color and shape).

#### Statistical analyses.

We used the same statistical procedure as experiment 2, analyzing separately Test 1 and Test 2.

### Results

**Fig 6:**
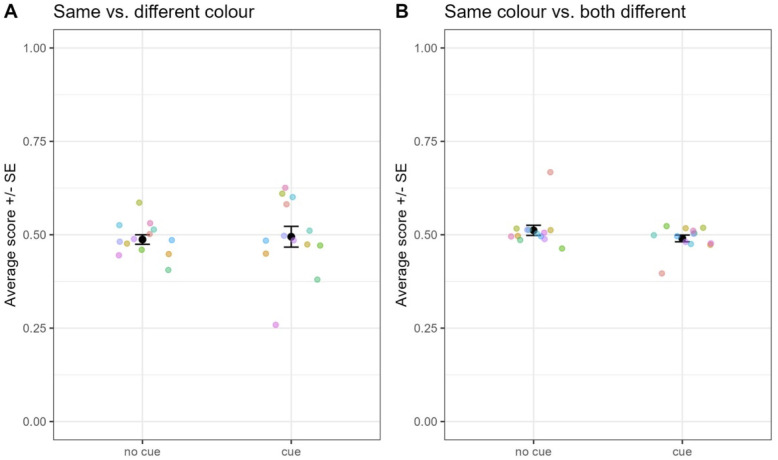
Performance in Experiment 3. This Fig shows the performance in the absence or presence of cue, when monkeys must match on the same vs. different color (Test 1: A), or on the same color vs. a different shape and color (Test 2: B). Legend as in Fig 4.

During Test 1 (Fig 6), we found no difference in performance in the absence of cue (match on the same color) compared to the presence of cue (match on different color; GLMM: β = 0.03, SE = .09, z = 0.33, p = 0.75) and both were at chance levels (no cue: β = −0.05, SE = .09, z = −0.59, p = 0.56; cue: β = −0.02, SE = .09, z = −0.33, p = 0.81).

Regarding Test 2, we found no difference in performance in the absence of cue (match on the same color) than with cue (match on different color and shape; β = −0.01, SE = .10, z = −0.12, p = 0.91) and both were at chance levels (no cue: β < 0.001, SE = .07, z = 0.00, p = 1; cue: β = −0.01, SE = .07, z = −0.17, p = 0.87).

### Interim discussion

In Experiment 3, the baboons again failed to show any understanding of the rule. The results were similar for Test 1, where the stimuli differed from the sample in only one dimension (color), and for Test 2, where they differed from the sample in both form and color.

All Experiments 1–3 used generalization as a test of rule learning. Being unable to demonstrate generalization, we explored in Experiment 4 an alternative possibility, which is that the baboons would show a relearning advantage when exposed to a simple version of the task involving a single cue, as was done in [17].

## Experiment 4

While Experiment 2 and 3 were very similar to what Dautriche et al. [17] did, they differed in using generalization rather than relearning to assess rule retention. In Experiment 4, we used relearning as a test.

### Materials and methods

#### Participants, living conditions and procedure.

The baboons, living conditions and test apparatus were the same as for Experiments 1–2.

Experiment 4 used two independent groups of baboons which were as much as possible counterbalanced for age and gender. One first group will hereafter be referred to the Consistent group (N = 9, 3 males, mean age = 16.5 years), the other will be referred to as the Inconsistent group, because it will be exposed to a reversal of the rule during testing (N = 10, 4 males, mean age = 13.4 years).

#### Test procedure.

**Fig 7:**
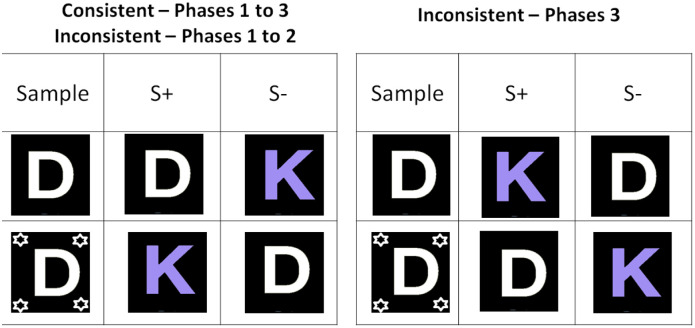
Task and general experimental design (Experiment 4).

The two groups of baboons (consistent and inconsistent) were each exposed to three test phases. The matching rule for the three phases of Consistent group, as well as for phases 1 and 2 of Inconsistent group is illustrated on the left on Fig 7. In that case the baboons had to identity match in absence of cue but had to provide a non-identity-matching response when the negative cue (the same as in Experiment 2) was present. Each phase consisted of blocks combining cued and non-cued trials and they were repeated until the learning criterion was reached. Critically, this matching rule was reversed in the last testing phase of the Inconsistent group, as illustrated on the left of Fig 7. This general procedure allows two main predictions if the baboons process the cue as a negation symbol. First, learning speed should get faster from phases 1–3 in the Consistent group, as well as from phase 1–2 in the Inconsistent group, because the same rule is applied from phase to phase. Second, the learning speed of the Inconsistent group should be much slower in phase 3 than for the consistent group, due to the rule reversal.

The left part of the figure shows the prototypical design used in the three phases of the Consistent group, and in phases 1 and 2 of the Inconsistent group. In that case, the baboons had to identity match in absence of cue, and to reverse the matching rule when the negation cue was present. The right part of the figure shows the rule applied to the last testing phase of the Inconsistent group. Note that four novel stimuli fully different in color and shape were assigned to each subject at the beginning of each test phase. The stimulus set was thus different from phase to phase, and among the subjects.

At the beginning of each phase, and independently for each subject, the test program randomly selected four stimuli per subject from a set of 100 possible stimuli made from the combination of 10 possible shapes (mostly numbers or letters) and 10 possible colors. The selection rule implied that the four stimuli within the prescribed set were all different in color and shape, and different from the stimuli already selected in the previous test phases. Once selected, this 4-item stimulus set was repeatedly used within the test phase until its end. The stimulus set used within each phase therefore varied between phases and differed from subject to subject

Each phase consisted in the randomized presentation of 48-trial blocks (24 cues and 24 non-cued trials) which used the stimulus arrangement shown in Fig 7. These blocks resulted from the counterbalancing of the cue condition (cued vs non cued), stimulus identity (4 possible stimuli per set), identity of the foil (the 3 alternative stimuli of the set), and the location of the target on the response display (left or right). The 48 trials blocks were repeated in a random order until the baboon reached 80% correct in the cued and non-cued condition. The dependent variable was the number of block repetitions required to reach the learning criterion. We further decided to stop training in phase 3 if the baboons failed to reach the learning criterion within 90 block repetitions.

#### Statistical analyses.

We used lme4 and lmerTest packages in R to construct linear mixed models to analyze the number of blocks needed to reach criterion, using the same random factor as before (individual identity) and studied the interaction between phase (categorical variable) and group (Consistent and Inconsistent).

### Results

**Fig 8:**
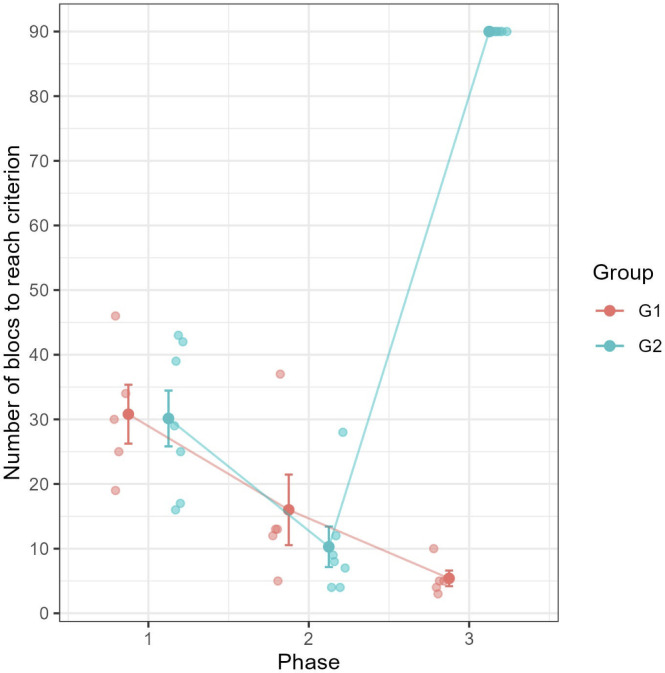
Number of blocks to reach learning criterion in the three phases of Experiment 4, and for groups 1 and 2. Transparent dots represent individual data points, opaque points the group mean and error bars the standard error.

Of the 19 individuals, seven did not finish all three phases of the experiment and were excluded from analysis (four from the consistent group, three from the inconsistent one, leaving a total of five and seven subjects respectively). As expected we found a learning effect between phase 1 and 2 (Fig 8): performance increased in both groups between the two phases (LMM Consistent group: β = −14.8, SE = 5.46, t = −2.71, p = 0.01; Inconsistent group: β = −19.9, SE = 4.62, t = −4.3, p < 0.001) and there was no difference between the two groups in each phase (phase 1: β = −0.66, SE = 5.06, t = −0.13, p = 0.90; phase 2: β = −5.71, SE = 5.06, t = −1.13, p = 0.27). When the consistent group is considered, performance continued to increase between phase 2 and 3, though this effect only approached significance (β = −10.6, SE = 5.46, t = −1.94, p = 0.06). By contrast, a sharp and significant decrease in performance was observed for the Inconsistent group (β = 79.7, SE = 4.62, t = 17.3, p < 0.001). In fact, none of the baboons from the Inconsistent group reached the learning criterion within 90 block repetitions, a clear and significant difference with the consistent group (Mann-Whitney U test, U = 0, p = 0.006).

### Interim discussion

In Experiments 1–3, we tested the capacity of baboons to learn the matching rule through generalization. They failed to generalize. In Experiment 4, which was essentially a replication of [17], we tested the baboons on relearning. Comparison between phases 1 and 2 revealed a performance improvement in phase 2 for both groups. An additional improvement which, only approached significance, was further found in phase 3 for the Consistent group, the group for which the matching rule remained the same as in training. By contrast, baboons in the Inconsistent group were drastically impaired in phase 3, when the matching rule was reversed in comparison to training. Altogether, their results clearly show a relearning advantage from phase to phase, when the same rule was maintained across phases.

### General discussion

To sum up, Experiment 1, which involved two cues and two dimensions, provided no evidence of generalization. Based on the hypothesis that using two cues and two dimensions made the task too complex for baboons, we conducted Experiments 2 (one cue, two dimensions) and 3 (one cue, one dimension — one cue, two dimensions), which progressively reduced the task complexity while still assessing generalization. Again, we found no evidence of generalization. In Experiment 4, which was similar to Experiment 2, we tested for relearning rather than generalization. We found evidence of relearning, confirming the results of [17].

These results, both negative and positive, demonstrate the limits of relearning. First, while no evidence of relearning was found in Experiment 1, Experiment 4 which involved a simpler version of the task did show relearning, suggesting that task complexity may hinder this process. The origin of this limitation remains unknown. It might be due to attentional or memory limitations. The second limit has to do with the fact that cognitive mechanisms of generalization and relearning are distinct, meaning that evidence of relearning is not a reliable indicator of generalization. Despite the task similarities between Experiment 2 and Experiment 4, there was no generalization in the first, but there was relearning in the second.

To discuss these results, it should firstly be recalled that matching and generalization were both previously demonstrated in baboons, suggesting that these are not, as such, the problem. Matching-to-sample is a standard task in animal cognition experimentation and is quite familiar to the baboon population we tested [22–23]. In addition, baboons have already manifested generalization when tested with perceptual [24–25], and conceptual [26] versions of the matching-to-sample task, as well as in other kinds of tasks, such as the odd-one out task [27], or sequence learning [28]. We further note that the baboons have already demonstrated their ability to process the shape and color dimensions of visual stimuli, independently from each other [29], and therefore that the ability to shift attention from one dimension to the other is not an issue either.

This raises the question of what exactly baboons are learning, and why generalization failed in our task, while relearning was found in experiment 4. Four hypotheses at least can be proposed to account for these results.

One can first suggest that the baboons did learn the meaning of the cues, but that the attraction of iconicity and novelty distracts them from referring to the rule they have learnt for responding. This would affect generalization but not relearning. Previous studies from our research group have demonstrated that novelty [30] and iconicity [17] can affect baboons processing of visual stimuli in matching tasks.

A second hypothesis is that baboons learn the rule (semantic compositionality). However, since compositionality alone is not sufficient to ensure productivity, we see evidence of relearning in Experiment 4, but no signs of generalization in Experiments 2 and 3. This could be explained by the fact that to go beyond compositionality and reach productivity (generalization), baboons would need much more training. If this is so, the failure of baboons to generalize in Experiments 2 and 3 would be a methodological artifact: while the training baboons underwent was sufficient for compositionality (as shown by relearning in Experiment 4), it was not sufficient for productivity (as shown by the failure of generalization in Experiments 2 and 3).

The third hypothesis accounting for the failure of generalization in Experiments 1–3 is that baboons do not extract the cue’s meaning but instead rely on rote memorization of each specific cue-sample pairing. Previous studies have demonstrated that baboons can learn by rote thousands of stimulus-response associations [31]. Although rote learning is in the scope of baboons, one could object that the evidence of relearning in Experiment 4 is a counterargument to this hypothesis of learning by rote. However, relearning in Experiment 4, in the absence of generalization in Experiments 2–3, might be due not to an understanding of the meaning of the cue, but rather to cognitive preparation due to the familiarity of the cue. In other words, this rote-learning process is accompanied by some sort of shallow statistical learning, insufficient however for attributing independent meaning to the cue, and, hence, for generalization. If this is the case, relearning in Experiment 4 would not contradict the hypothesis according to which all cue-sample combinations are learnt by rote.

Finally, the fourth hypothesis involves a distinction between implicit and explicit memory: generalization may require some forms of explicit processing of the rule, engaged by the task, whereas relearning may reflect more implicit learning—possibly based on the statistical regularities of the task. Unfortunately, none of the four hypotheses outlined above can be definitively confirmed or ruled out based on the data currently available.

### Conclusion

We were interested in whether baboons were capable of semantic compositional processing within the framework of the task. If semantic compositionality is seen as entailing productivity (tested through generalization), the results presented here do not allow us to claim that they are capable of semantic compositionality. By contrast, if semantic compositionality is dissociated from productivity [32–33], the lack of generalization observed in Experiments 2 and 3 could be due to either a methodological issue (insufficient training) or limitations within the baboons’ cognitive system. Under these circumstances, the evidence of relearning in Experiment 4 might suffice to demonstrate that baboons are capable of semantic compositionality, even if they cannot generalize the compositional rule to new items.

## Supporting information

S1 Table Procedural table for all four experiments. (DOCX)

S2 Table Results of the preliminary test of Experiment 2. (DOCX)
