# Supplementary material for: Testing semantic compositionality in baboons (Papio papio) through relearning and generalization
Source: PLoS One. 2025 Nov 5;20(11):e0334726. doi: 10.1371/journal.pone.0334726 (PMC12588492; doi:10.1371/journal.pone.0334726)
Supplement: S1 Table — (DOCX) [file pone.0334726.s001.docx]

**Table S2 : Procedural details**

|  | **Experimental phase** | **Number of subjects** | **Number of cues** | **Number of colors** | **Number of shapes** | **Number of trials per block** | **Training criterion** |
| --- | --- | --- | --- | --- | --- | --- | --- |
| Experiment 1 | Phase 1 | 23 | 2 | 2 | 2 | 120 (60 color and 60 shape trials) | 66% within a block for both color and shape trials |
|  | Phase 2 |  | As above | 3 (2 old +1new) | 3  (2 old +1new) | 120 - 30 per condition (old-color, old shape, new color, new shape trials) | 66% in each condition (n=4) |
|  | Phase 3 |  | As above | 4 (3 old + 1 new) | 4 (3 old + 1 new) | As above | As above |
|  | Phase 4 |  | As above | 5 (4 old + 1 new) | 5 (4 old + 1 new) | As above | As above |
|  | Phase 5 |  | As above | 6 (5 old + 1 new) | 6 (5 old + 1 new) | As above | As above |
|  | Phase 6 |  | As above | 7 (6 old + 1 new) | 7 (6 old + 1 new) | As above | As above |
|  | Phase 7 |  | As above | 8 (7 old + 1 new) | 8 (7 old + 1 new) | As above | As above |
|  | Phase 8 |  | As above | 9 (8 old + 1 new) | 9 (8 old + 1 new) | As above | As above |
|  | Phase 9 |  | As above | 10 (9 old + 1 new) | 10 (9 old + 1 new) | As above | As above |

| Experiment 2  (Preliminary) | Training | 22 | None | 2 | 2 | 64 (8 per stimulus : n=4) | 80% |
| --- | --- | --- | --- | --- | --- | --- | --- |
|  | Testing |  | None | 2 | 2 | 72 (64 training + 8 test trials). The block is repeated 5 times | None |
| Experiment 2  (Main) | Testing | 22 | 1 (negation cue) | 10 (8 novel + the above 2) | 10 (8 novel + the above 2) | 768 trials (648 training trials with the just above color and shape, 50% cued, 50% non-cued), mixed with 64 trials with novel colors and shapes (50% cues and 50% non-cued) | None |
| Experiment 3 | Training | 22 | 1 (negation cue) | 2 | 2 | 96 (48 cued and 48 non-cued trials) | 80% |
|  | Test 1 |  |  | 8 (2 old as in training + 6 novel colors) | 8 (2 old as in training + 6 novel shapes) | 1104 (960 identical to training mixed with 72 test trials involving 6 novel colors and 6 novel Shapes) | None |
|  | Test 2 |  |  | 2 old as in training + 6 other novel colors) | 2 old as in training + 6 other novel colors) | 1104 (960 identical to training mixed with 72 test trials involving 6 novel colors and 6 novel Shapes) | None |

| Experiment 4 | Phase 1  (Group Consistent and Inconsitent) | 19 | 1 (negation cue) | 4 (all stimuli differ in color) | 4 (all stimuli differ in shape) | 48 (24 cued and 24 non-cued trials). The block is repeated until the criterion is reached | 80% |
| --- | --- | --- | --- | --- | --- | --- | --- |
|  | Phase 2  (Group Consistent and Inconsitent) |  | As above | As above | As above | A above | 80% |
|  | Phase 3 (Consistent group) |  | As above | As above | As above | As above | 80% |
|  | Phase 3 (Consistent group) |  | As above | As above | As above | Same design as above but the matching rule is reversed for this group | 80% |
