# Supplementary material for: Testing semantic compositionality in baboons (Papio papio) through relearning and generalization
Source: PLoS One. 2025 Nov 5;20(11):e0334726. doi: 10.1371/journal.pone.0334726 (PMC12588492; doi:10.1371/journal.pone.0334726)
Supplement: S2 Table — (DOCX) [file pone.0334726.s002.docx]

Supplemental information, Table S1

|  | N color | N form | N trial | Frequency (color) | CI low | CI upp | pval | adj pval |
| --- | --- | --- | --- | --- | --- | --- | --- | --- |
| MALI | 39 | 1 | 40 | 0.98 | 0.868 | 0.999 | 0 | 0 |
| ARIELLE | 37 | 3 | 40 | 0.93 | 0.796 | 0.984 | 0 | 0 |
| LIPS | 37 | 3 | 40 | 0.93 | 0.796 | 0.984 | 0 | 0 |
| FEYA | 36 | 4 | 40 | 0.90 | 0.763 | 0.972 | 0 | 0 |
| MAKO | 36 | 4 | 40 | 0.90 | 0.763 | 0.972 | 0 | 0 |
| HARLEM | 35 | 5 | 40 | 0.88 | 0.732 | 0.958 | 0 | 0 |
| LOME | 35 | 5 | 40 | 0.88 | 0.732 | 0.958 | 0 | 0 |
| EWINE | 34 | 6 | 40 | 0.85 | 0.702 | 0.943 | 0 | 0 |
| NEKKE | 34 | 6 | 40 | 0.85 | 0.702 | 0.943 | 0 | 0 |
| PETOULETTE | 34 | 6 | 40 | 0.85 | 0.702 | 0.943 | 0 | 0 |
| PIPO | 34 | 6 | 40 | 0.85 | 0.702 | 0.943 | 0 | 0 |
| ANGELE | 33 | 7 | 40 | 0.82 | 0.672 | 0.927 | 0 | 0 |
| FELIPE | 33 | 7 | 40 | 0.82 | 0.672 | 0.927 | 0 | 0 |
| FLUTE | 33 | 7 | 40 | 0.82 | 0.672 | 0.927 | 0 | 0 |
| VIOLETTE | 33 | 7 | 40 | 0.82 | 0.672 | 0.927 | 0 | 0 |
| MUSE | 32 | 8 | 40 | 0.80 | 0.644 | 0.909 | 0 | 0 |
| DORA | 30 | 10 | 40 | 0.75 | 0.588 | 0.873 | 0.002 | 0.002 |
| FANA | 29 | 11 | 40 | 0.73 | 0.561 | 0.854 | 0.006 | 0.007 |
| ARTICHO | 27 | 13 | 40 | 0.68 | 0.509 | 0.814 | 0.038 | 0.042 |
| ATMOSPHERE | 26 | 14 | 40 | 0.65 | 0.483 | 0.794 | 0.081 | 0.085 |
| DREAM | 18 | 22 | 40 | 0.45 | 0.293 | 0.615 | 0.636 | 0.636 |

Result of the preliminary test of Experiment 2.

N color = Number of color responses

N form = Number of form responses

N trial = Number of test trials.

Frequency color = mean frequency of color responses

CI low = confidence interval low boundary

CI high = confidence interval high boundary

Pval

Adj pval
